# Close 3D proximity of evolutionary breakpoints argues for the notion of spatial synteny

**DOI:** 10.1186/1471-2164-12-303

**Published:** 2011-06-10

**Authors:** Amélie S Véron, Claire Lemaitre, Christian Gautier, Vincent Lacroix, Marie-France Sagot

**Affiliations:** 1 Université de Lyon, F-69000 Lyon, France; 2 Laboratoire Biométrie et Biologie Evolutive, CNRS, Université Lyon 1, F-69100 Villeurbanne, France; 3 Equipe BAMBOO, INRIA Grenoble Rhône-Alpes, 655 avenue de l'Europe, F-38330 Montbonnot Saint-Martin, France; 4 INSERM U1052, Cancerology Research Center of Lyon, Centre Léon Bérard, Lyon, France; 5 Université de Bordeaux, Centre de Bioinformatique et Génomique Fonctionnelle Bordeaux, F-33000 Bordeaux, France; 6 Equipe SYMBIOSE, INRIA Rennes Bretagne Atlantique, Campus de Beaulieu, F-35042 Rennes, France

## Abstract

**Background:**

Folding and intermingling of chromosomes has the potential of bringing close to each other loci that are very distant genomically or even on different chromosomes. On the other hand, genomic rearrangements also play a major role in the reorganisation of loci proximities. Whether the same loci are involved in both mechanisms has been studied in the case of somatic rearrangements, but never from an evolutionary standpoint.

**Results:**

In this paper, we analysed the correlation between two datasets: (i) whole-genome chromatin contact data obtained in human cells using the Hi-C protocol; and (ii) a set of breakpoint regions resulting from evolutionary rearrangements which occurred since the split of the human and mouse lineages. Surprisingly, we found that two loci distant in the human genome but adjacent in the mouse genome are significantly more often observed in close proximity in the human nucleus than expected. Importantly, we show that this result holds for loci located on the same chromosome regardless of the genomic distance separating them, and the signal is stronger in gene-rich and open-chromatin regions.

**Conclusions:**

These findings strongly suggest that part of the 3D organisation of chromosomes may be conserved across very large evolutionary distances. To characterise this phenomenon, we propose to use the notion of spatial synteny which generalises the notion of genomic synteny to the 3D case.

## Background

In the last decade, our view of genome organisation started to greatly change once again with the realisation that the spatial arrangement of eukaryotic chromosomes inside cells is not random. Such arrangement was called the nuclear architecture by Cremer and Cremer, who showed that during interphase, chromosomes seem to occupy distinct territories with preferential locations relative to the nuclear center [1]. Spatial proximity between genetic elements situated at distant positions along the genome or even on different chromosomes is known to be important for gene expression. For instance, transcription seems to be localised within discrete regions that have been called "transcription factories" [2,3]. Those are multifunctional supercomplexes able to process several, often distally located genes. More recently, spatial proximity was shown to also correlate with translocation frequencies in somatic cells, including across different chromosomes [4]. This work provided evidence that chromosome territories may intermingle.

**Figure 1:**
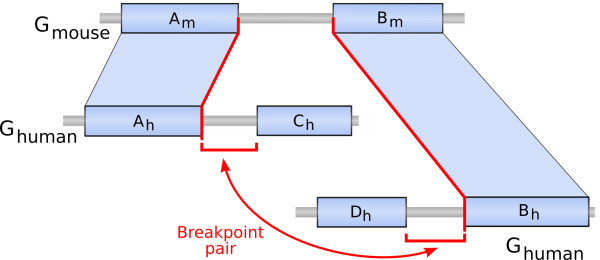
**Grouping breakpoints by pairs**. Schematic representation of a breakpoint pair. Parts of the human and mouse genomes are represented with synteny blocks drawn as blue rectangles and the breakpoints are the regions between two consecutive synteny blocks. The breakpoint (*A_m _*- *B_m_*) located on the mouse genome is flanked by two synteny blocks, *A *and *B*, which are not consecutive on the human genome. It is thus orthologous at its extremities to two breakpoints on the human genome flanking the two blocks *A *and *B*: (*A_h _*- *C_h_*) and (*D_h _*- *B_h_*). These two human breakpoints, represented by the red segments, can then be grouped in a pair and correspond to regions that are adjacent on the mouse genome.

Taking this observation one step further, we ask here whether chromatin interactions are correlated with genomic rearrangements that are conserved throughout evolution. We detected breakpoint regions (Figure 1) resulting from evolutionary rearrangements which occurred since the split of the human and mouse lineages using a method we previously developed [5]. We obtained a set of region pairs that are genomically distant in the human lineage, but adjacent in the mouse linage. We refer to these human genomic regions as breakpoint pairs.

To study the spatial (3D) proximity of these regions in the human lineage, we used the first and so far only whole-genome proximity map available for human cells [6]. These maps were obtained using Hi-C, a method that identifies chromatin interactions across an entire genome by coupling proximity-based ligation with massively parallel sequencing. While Hi-C is a complex experimental procedure, it can be thought of as a means to quantitatively sequence pairs of DNA fragments that were in close 3D proximity in live cells (for more details, see [6]).

Our purpose in comparing these two datasets is to test whether loci which are genomically distant in the human genome but adjacent in mouse, tend to be brought close to each other through 3D chromatin folding in human cells. This would argue in favour of a conservation of spatial proximities over large evolutionary distances and support the notion of spatial synteny. Moreover, this would also give evidence of a conservation of spatial proximities across cell types since we are using a proximity map which was established in a lymphoblastoid cell line while the rearrangements we study occurred in the germline and a affected all cell lines.

In the first part of this paper, we ask whether pairs of loci containing breakpoint-region pairs are more frequently observed to be in spatial proximity than other locus pairs at a similar genomic distance in human. We then check if the lineage of origin of a breakpoint pair or its evolutionary re-use has an influence on the 3D proximity of the pair. For all these questions, we controlled for both biological and methodological confounding factors. Finally, and more generally, we show that the method used to map short reads to repetitive regions of a genome has to be chosen with extreme caution when dealing with Hi-C data, as this may lead to an over-estimation of the frequency of interaction of distant loci containing repeats.

## Results

In this analysis, we divided the human genome in non overlapping windows (or loci) of 1 Mb and compared the three-dimensional (3D) proximity of pairs of loci containing or not a breakpoint pair. As suggested in [6], we used the number of Hi-C read pairs between two 1-Mb genomic loci as a proxy for their 3D proximity. We detected breakpoints on the human genome using the software Cassis [7], and grouped them by pairs such that each distant pair in the human genome corresponds to adjacent loci in the mouse genome (see Figure 1 and Methods). As a consequence, human locus pairs containing a pair of breakpoints constitute a subset of the human genome that is genomically distant in human and genomically close in mouse. We obtained 294 locus pairs containing at least one breakpoint pair. Among them, 126 are inter-chromosomal and 168 are intra-chromosomal.

### Pairs of loci containing breakpoint pairs tend to be close in 3D in human cells

The major result of the paper is that pairs of loci containing breakpoint pairs are significantly closer in 3D than pairs of loci at similar genomic distances but not containing breakpoint pairs. We show that this result can be explained neither by confounding factors, nor by methodological biases introduced at the mapping stage.

#### Controlling for possible confounding factors

##### Genomic distance

**Figure 2:**
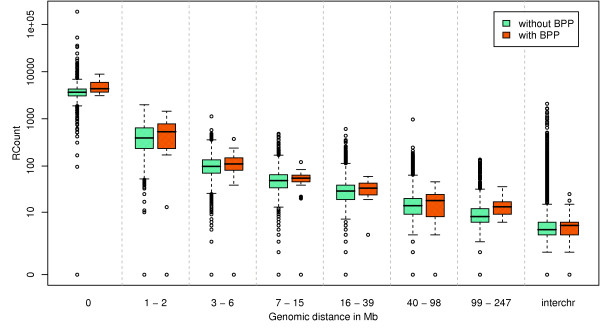
**Frequency of interaction of locus pairs containing or not breakpoint pairs**. Frequency of interaction (read counts) of locus pairs containing or not breakpoint pairs (BPP) in several classes of genomic distances. The two axis are in log scale. The read count is corrected for the presence of segmental duplications and assembly gaps.

First, we observe that the further two loci are on one chromosome, the less often they are in 3D proximity, with a linear decrease in the log scale (Figure 2, Additional file 1, Figure S1). This is expected and is not a new result. However, it is crucial to model explicitly the relation between genomic proximity and spatial proximity because pairs of loci containing breakpoints are not randomly distributed on the genome. Instead, they are closer to each other than the remaining of the locus pairs (median of 5.5 Mb vs. 48 Mb). When testing for a correlation between breakpoints and spatial proximity, we therefore need to control for genomic distance as a possible confounding factor.

We assessed the effect of confounding factors using Analysis of Covariance (ANCOVA) by comparing embedded models where factors are added one by one. For each pair of embedded models, the significance of the target parameter in the model (in our case the presence of breakpoint pair) is tested (see Methods for more details about the models used). Taking into account the fact that the further two loci are on the genome, the less they interact, we find that loci containing breakpoints are significantly closer in 3D than loci not containing breakpoints (*M*_1 _vs. *M*_0_, *p *= 4.74 * 10^-06^). Importantly, this holds whatever the distance separating the loci on a same chromosome (Figure 2). We further notice that the difference is stronger for short distances (*M*_2 _vs. *M*_1_, *p *= 5.61 * 10^-13^), which is also the class of distance for which we have more data, hence more statistical power. The first bin of distances can also be modelled separately and, again, shows the same trend for all distances within the first Mb (Additional file 1, Figure S2). Interestingly, we notice that, although they do exist, inter-chromosomal contacts are less frequent than very distal intra-chromosomal contacts. We also tested for a difference between loci containing or not breakpoint pairs among inter-chromosomal locus pairs, but the difference was not significant (Wilcoxon Rank-sum test, *p *= 0.48).

##### Gene density and state of chromatin

We now ask whether the location of breakpoints in specific regions of the genome could explain their higher 3D proximity. Indeed, breakpoints are located preferentially in gene-rich and open-chromatin regions [8], which we expect will themselves have a higher level of chromatin interactions than the remaining of the genome. We thus investigated the relations between 3D proximity and two potentially confounding variables: gene density and sensitivity to the enzyme DNaseI (taken as a proxy of chromatin state) in each locus. We notice that DNase sensitivity and gene density explain part of the variance of 3D proximities: pairs of loci having more genes and that are more sensitive to DNaseI (in an open chromatin state) tend to be closer in 3D.

We then tested if, when either the gene density or the state of chromatin of each locus is taken into account, we still see a difference between pairs of loci containing or not containing a breakpoint pair. We can still see a difference, which is even stronger in regions containing more genes and regions more sensitive to DNase (ANCOVA results, *M*_5 _vs. *M*_4_, *p *= 0.030 for gene density; *M*_8 _vs. *M*_7_, *p *= 6.34*e *- 11 for DNase sensitivity, see Methods for more details, as well as Additional file 1, Figures S3 and S4). Hence, especially among the gene-rich and open chromatin regions, those containing breakpoints are closer in 3D than pairs of loci not containing breakpoints.

##### Simulations confirm previous results

**Figure 3:**
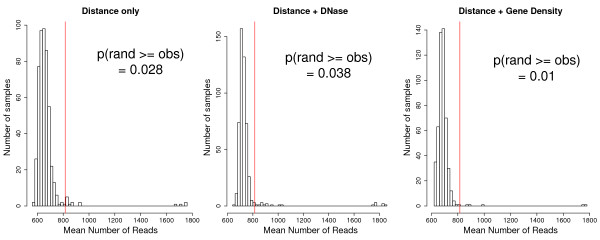
**Locus pairs containing a breakpoint pair have more reads than expected**. Histogram of values obtained by sampling 500 times the pairwise read count data, using pairs without breakpoints but at the same distance (left), same distance and same DNaseI sensitivity (middle), same distance and same gene density (right) as those in the breakpoint sets. The red vertical bar show the value for the actual breakpoint set.

We confirmed these results with simulations where the mean number of reads of a set of locus pairs without breakpoints but having the same characteristics (in terms of genomic distance, DNaseI sensitivity or gene density) as the locus pairs containing breakpoints is compared to the mean number of reads for breakpoint-containing locus pairs (Figure 3 and Methods). Again, we find that the inter-chromosomal locus pairs are not significantly closer when they contain a breakpoint (p = 0.2 when controlling for gene content, p = 0.22 when controlling for DNase sensitivity). Loci on the same chromosome are confirmed to be in closer 3D proximity when they contain a breakpoint pair (p = 0.03 when controlling for genomic distance only, p = 0.01 when controlling for gene density and genomic distance, p = 0.04 when controlling for DNase sensitivity and genomic distance).

#### Controlling for a possible methodological bias

**Figure 4:**
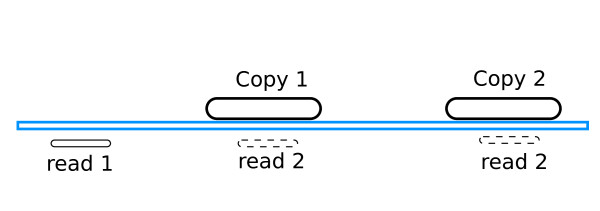
**Mapping of a read pair to a reference genome containing a repetitive sequence**. Mapping of a read pair to a reference genome. The first read of the pair maps to a unique location, the second read maps to a repetitive sequence, present in two copies in the genome. The read may therefore be assigned to two locations. The method used by the Dekker group considers that these two locations are equiprobable, whereas the first location should be preferred. This results in an overestimation of the interaction frequency between distant loci when at least one of the involved loci contains a repetitive sequence.

A central issue when dealing with short (35 bp) reads is that it is not always obvious to assign each read to a unique location in the reference genome. This is especially difficult when the fragment to be sequenced is part of a repetitive sequence. The read will in this case map to multiple locations. Lieberman *et al*. use for their mapping the MAQ software [9], which chooses one of the locations randomly. While this method is expected to perform well in the case of genome (re)-sequencing, it is not adapted to Hi-C data which is composed of read pairs, the two reads corresponding to two loci that are in 3D proximity [6]. Indeed, when one read of a read pair maps to a unique location and the other read maps to a repetitive sequence, it is a mistake to consider that all copies of the repetition are equiprobable mapping positions for the second read (Figure 4). The copy genomically closer to the first mapped read should be preferred, since loci that are close on the genome are expected to be closer in 3D than loci that are more distant.

A consequence of using MAQ [9] for mapping as done in [6] is that the physical interaction between distant loci will be overestimated every time one of the involved loci contains a repetitive sequence. To correct for this bias, we devised a simple method which discards reads mapped to a known segmental duplication and uses the number of reads mapping to the neighbourhood of repeats to estimate the true interaction between loci containing repetitions (see Methods). All figures and results presented in this paper were obtained with the read count corrected for repetitions.

### Impact of the mapping method on the subclassification of breakpoints

While taking into account mapping biases did not change our main conclusion, we now show that it does have a significant impact when we test further hypotheses related to the lineage of origin or the potential re-use of breakpoints. This indicates that it is crucial to take extreme caution when using readily available mapped data.

#### Breakpoints which occurred in the human lineage are not significantly closer in 3D than breakpoints which occurred in the mouse lineage

**Figure 5:**
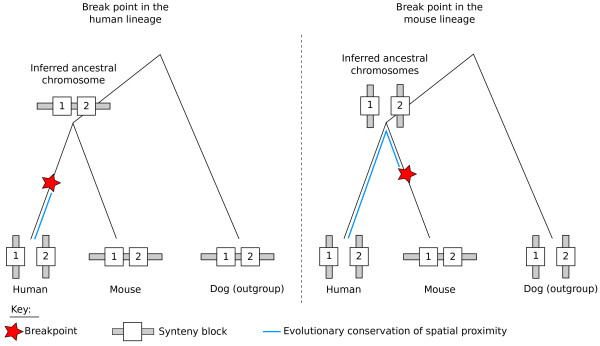
**Evolutionary conservation of spatial proximity**. Evolutionary conservation of spatial proximity up to the current human genome organisation depending on the lineage of origin of the breakpoint.

For each breakpoint pair, using the dog genome as an outgroup, we tried to assess whether the rearrangement occurred in the human (40) or in the mouse (115) lineage (see Methods and Figure 5). We were unable to assign an origin to the 139 remaining events.

We then tested if the origin of the break had any influence on the spatial proximity of the breakpoint pair. Our expectation was that human lineage breaks were kept closer in 3D than mouse lineage breaks since, for the former, the time of conservation of spatial proximity would be shorter (see discussion).

**Figure 6:**
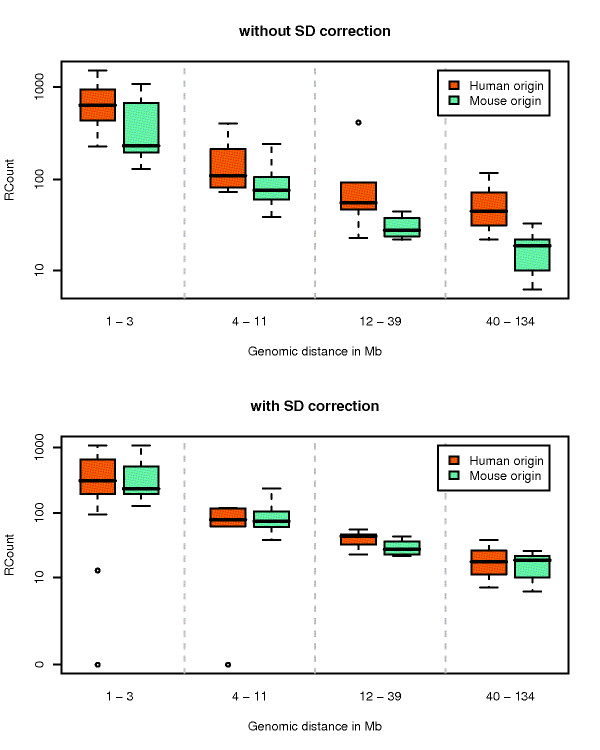
**Frequency of interaction of locus pairs containing breakpoint pairs of human or mouse origin**. Frequency of interaction (read counts) of locus pairs containing breakpoint pairs of human or mouse origin in several classes of genomic distances. In the upper figure, the read count is not corrected for the presence of segmental duplications.

Although we initially detected a significant difference between the two categories of breakpoints (ANCOVA, *p *= 2.15*e *- 06), we were unable to repeat this result after correcting for repeats (ANCOVA, *p *= 0.232) (Figure 6). Indeed, we observe that segmental duplications in the genome are very frequent in loci containing breakpoints of human lineage origin, as previously reported [10-12]. Since this trend is not observed in breakpoints of mouse lineage origin, correcting for segmental duplication mapping problems proved crucial in our analysis.

#### Reciprocal and Non-Reciprocal Breakpoints

A same genomic region can be re-used as a breakpoint for several rearrangements, either in the same lineage or in distinct lineages [13-15]. However, the cause for such "fragility" remains unexplained. Given that different mechanisms and/or evolutionary forces may be at play for re-used and non re-used breakpoint pairs, we asked whether there is any difference in terms of spatial proximity between these two types of breakpoints.

**Figure 7:**
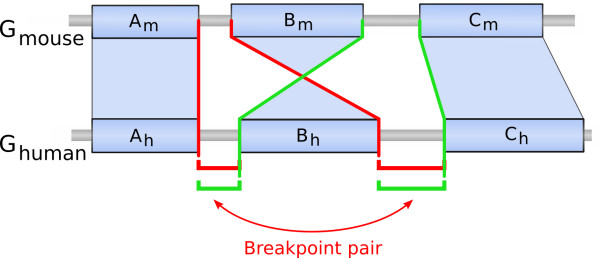
**Example of a reciprocal breakpoint pair**. Schematic example of a reciprocal breakpoint pair. In this schematic representation of two parts of the mouse and human genomes (blue rectangles represent synteny blocks), a simple rearrangement is represented: the inversion of the synteny block *B*. Following the definition of breakpoint pairs, looking at the mouse breakpoint (*A_m _*- *B_m_*), the two human breakpoints (*A_h _*- *B_h_*) and (*B_h _*- *C_h_*) are grouped together (in red). Then, looking at the mouse breakpoint (*B_m _*- *C_m_*), we can also group together the two human breakpoints (*A_h _*- *B_h_*) and (*B_h _*- *C_h_*) (in green). Thus these two human breakpoints cannot be grouped with any other breakpoint and are referred to as a reciprocal breakpoint pair.

**Figure 8:**
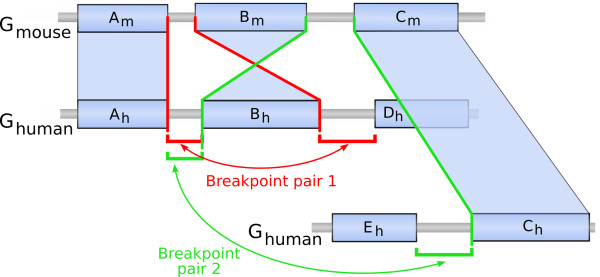
**Example of a non-reciprocal breakpoint pair**. Schematic example of a non-reciprocal breakpoint pair. In this schematic representation of two parts of the mouse and human genomes (blue rectangles represent synteny blocks), a complex series of rearrangements is represented: first the block *B *has been reversed and then the block *C *has been moved away; note that these two rearrangements use the same breakpoint (*B_m _*- *C_m_*) (it is a case of breakpoint re-use). Following the definition of breakpoint pairs, looking at the mouse breakpoint (*A_m _*- *B_m_*), the two human breakpoints (*A_h _*- *B_h_*) and (*B_h _*- *D_h_*) are grouped together (in red). On the other hand, looking at the mouse breakpoint (*B_m _*- *C_m_*), the breakpoint (*A_h _*- *B_h_*) is then grouped with the breakpoint (*E_h _*- *C_h_*) (in green). Since the breakpoint (*A_h _*- *B_h_*) can be involved in two distinct pairs, the latter pairs are thus called non-reciprocal.

In the pairing process, we can distinguish between breakpoint pairs resulting from a simple event (we call them reciprocal) and more complex series of events (called non-reciprocal) that are likely to involve re-use (see Methods and Figures 7 and 8). The evolutionary origin of most non-reciprocal breakpoint pairs remained elusive, highlighting the loss of evolutionary signal due to re-use. Introducing the dog genome as an outgroup, we obtained contradictory assignations for the diverse components of the breakpoint pairs. However, when comparing reciprocal and non-reciprocal breakpoint pairs, we could find no significant difference in terms of spatial proximity.

## Discussion

In this paper, we used whole-genome chromatin contact data from the Hi-C technology [6] to study the spatial behaviour of evolutionary breakpoints. We were able to show that loci which are distant in the human genome but adjacent in mouse are observed significantly more often in close proximity in the human nucleus than expected. This result holds for loci located on a same chromosome regardless of the genomic distance separating them.

Similar correlations were previously observed with other types of rearrangement events. Cancer and somatic translocations, as well as radiation-induced rearrangements depend on spatial proximity and intermingling within chromosome territories [4,16,17]. These correlations were explained by a mechanistic hypothesis suggesting that DNA sequences in close proximity are more likely to be part of a rearrangement (for a review see [18]). In mammals, experimental studies have shown that Double Strand Breaks (DSBs) are not mobile in the nucleus [19,20]. This argues in favour of a contact-first model where the loci involved in a rearrangement must be in spatial proximity before a mismatch repair between DSBs can happen.

The mechanistic scenario is able to explain the 3D proximity in the time immediately following a rearrangement. However, it is not sufficient to explain the conservation of spatial proximity over a longer period of time. In our case, we observe 3D proximity between loci that were rearranged at some point in the course of evolution since the last common ancestor of human and mouse, that is, potentially up to 80 million years ago. Although it had been previously reported that spatial organisation of the genome inside the nucleus is not random and that preferential interactions exist [1,21], our result not only confirms this previous report but also suggests that the time scale of the conservation may be much larger than anticipated.

Our result therefore also suggests that some 3D proximities are conserved across cell-types, as previously noticed [22,23]. Indeed, we observe a correlation between evolutionary events which happened in germline cells and the current 3D organisation in a lymphoblastoid cell line. The signal we detect is therefore indirect. In order to be maintained throughout evolution, these 3D proximities, or at least the information encoding these 3D proximities, had to be passed to the next generation, that is, had to be present in the germline. Establishing whether the 3D proximity itself, or only its encoding, is present in the germline would require to have a Hi-C dataset obtained in the germline. Since no such dataset is available, we cannot give a clear answer to this point. We could however argue that, under the contact-first model, since the loci had to be in close contact for the break to occur, it is more parsimonious to assume that they have been kept in contact ever since in the germline.

We observe that the time of conservation of the spatial proximity may be different depending on whether the break occurred in the human lineage or in the mouse lineage, as shown in Figure 5. If the rearrangement occurred in the human lineage (two genomically adjacent loci broke apart in the human lineage), it implies that the two loci were genomically close to each other in the ancestor and therefore also close in 3D. In order to explain the observed pattern, we need to invoke that the spatial proximity was maintained since the break. On the other hand, if the rearrangement occurred in the mouse lineage, the two loci were most likely not genomically close to each other in the ancestor. They are however observed today genomically close in mouse, and spatially close in human nuclei. If the parsimony principle applies, proximity was present in the ancestor. However, in this case, the proximity was not genomic, it was spatial. Overall, the spatial proximity in this case was conserved over more than 80 million years (from the speciation to human, and from the speciation to the break in mouse, see Figure 5).

In this study, we were able to observe the correlation of 3D proximities with intra-chromosomal breakpoint pairs only. The lack of signal for inter-chromosomal loci could be explained by the overall lower number of inter-chromosomal reads (5 reads on average per locus pair), which reduces the power of statistical analyses. Alternatively, inter-chromosomal interactions could be less conserved through evolution or cell-types. The notion of chromosome territories corroborates this hypothesis since intra-chromosomal proximities are constrained inside a defined territory. In addition, chromosome territories are found in different cell-types and species but their arrangement with respect to each other seems to differ between cell-types and species [24].

The function of long-range chromatin interactions and the mechanisms responsible for their maintenance are still mostly unknown and go beyond the scope of this paper. We may however observe that several hypotheses can be formulated to address this point. We can for instance mention the participation of loci to the same transcription factory [22,25,26], their synchronisation during replication [27], or the fact that they bind to a common protein, such as CTCF [28]. Although it does not enable to clearly favour one of these hypotheses, our result sheds a new light on this field by bringing evidence that long-range chromatin interaction loci are enriched in evolutionary breakpoints. In the case of a rearrangement separating two loci, 3D proximity could represent a functional compensation for the loss of genomic synteny. Indeed, the arrangement of genes and of functional regions is not random along the chromosomes, and positional changes due to rearrangements may have serious consequences on the fitness of an organism. If the two separated loci need, for instance, to be co-expressed, the rearrangement will probably not be selected, unless the loci can be brought next to each other in the cell by another means, for instance through chromatin interactions at transcription factories. We therefore suggest the existence of a spatial synteny, which is another level of organisation of the genes, in addition to genomic synteny. The fact that we found a stronger correlation in gene-rich and open-chromatin regions argues at least for a gene or transcription-dependent mechanism maintaining 3D proximities. Indeed, if 3D proximities are driven by active gene and transcription factories, we can expect the signal to be stronger in gene-rich regions.

In this paper, we were able to show that some 3D proximities may be maintained over a long period of time. We also show that the loci which are maintained in contact lie within open-chromatin, gene-dense regions, and contain breakpoint pairs. These 3 features are therefore all related to the conservation of the spatial arrangement of loci, but it remains an open question which of these features, if any, is the driver. We were able to rule out gene density and DNaseI sensitivity as possible confounding factors in our analysis and showed that taking into account these factors, breakpoint pairs are still closer in 3D than other locus pairs. However, we may have missed another unknown genomic feature which could also explain the close proximity of breakpoints. Nevertheless, even if spatial proximity was not directly a cause or a compensation for the rearrangement, it remains to be explained why rearrangements occur in these specific regions, and why these regions are maintained close in 3D through evolution.

Finally, on the methodological side, we outlined a point related to the treatment of repetitive sequences which has been crucial in the analysis and goes much beyond the context of this paper. We showed that the mapping method used in the Hi-C publication over-estimates the 3D proximity of distant loci when at least one locus contains a repetitive sequence. Our own way of compensating for these repetitive sequences might be too conservative and under-estimate the interaction between distant loci containing repeats. Although weaker, the correlation between breakpoints and 3D proximities was still significant after our correction and would only benefit from a more specific correction method. However, it may be that the conclusions w.r.t. the origin of the breakpoint could change if we are indeed under-estimating the true interaction intensity between distant copies. Dedicated methods and/or technologies that enable to deal with the mainly methodological-related difficulties of handling such regions are therefore needed.

## Conclusion

In this paper, we analysed the spatial proximity in the nucleus of loci involved in a rearrangement between human and mouse. We considered breakpoints resulting from any type of evolutionary rearrangement, which we detected with a method that we previously developed. We showed that two loci distant in the human genome but adjacent in the mouse genome are significantly more often observed in close proximity in the human nucleus than expected, and this result holds for loci located on the same chromosome regardless of the genomic distance separating them. These findings strongly suggest that part of the 3D organisation of chromosomes may be conserved across very large evolutionary distances. To characterise this phenomenon, we propose to use the notion of spatial synteny which generalises the notion of genomic synteny to the 3D case.

## Methods

### Sequence and annotation data

The breakpoint data were obtained by comparing the genomes of human and mouse using Cassis [5,7]. Sequences, annotations and orthologous gene relationships were retrieved from the Ensembl genome browser, release 54 [29]. The following assembly releases were used: human assembly of November 2005 (NCBI36 or hg18), mouse assembly of April 2007 (NCBIM37 or mm9) and dog assembly of May 2005 (CanFam2.0).

### Defining the breakpoints

Cassis is a method to precisely localise breakpoints on one genome compared to the genome of a related species. It is composed of two steps: i) in the first, synteny blocks and breakpoints are identified, ii) in the second, breakpoint regions are refined in one genome. Synteny blocks are pairs of orthologous regions which have not been rearranged between the two genomes. They are identified by comparing the order and orientation of sets of orthologous genes (for details, see [5]). Breakpoint regions are then defined as regions between two consecutive synteny blocks on one genome whose orthologous blocks on the other genome are not consecutive, or not in the same orientation. In the second step, each breakpoint on one genome is refined by aligning its sequence against its orthologous sequences on the other genome. A segmentation algorithm computes the new coordinates of the breakpoint based on the alignments.

We applied Cassis to the human and mouse genomes and identified breakpoints on the two genomes. To improve the resolution of our dataset, each breakpoint region on the human genome was then refined using the second step of Cassis. We obtained 373 breakpoints with a median size of 54 Kb mapped on the human genome.

### Grouping breakpoints by pairs

Since a breakpoint is flanked by two synteny blocks which are not adjacent (or not in the same orientation) on the other genome, each breakpoint region on one genome is orthologous at its extremities to two breakpoint regions on the other genome (see Figure 1). Notice that telomeres (extremities of chromosomes) can be considered as breakpoints, even if they are not strictly speaking located between two synteny blocks. Thus for each breakpoint region on the mouse genome, we can link together two human breakpoints. The latter pair corresponds to regions which are apart on the human genome but adjacent on the mouse. We can infer that, either they were separated by a rearrangement in the human lineage and used to be adjacent before, or they were placed adjacent to one another in the mouse lineage due to a rearrangement in this lineage.

We distinguished two types of breakpoint pairs: the reciprocal ones and the others. Breakpoints in reciprocal pairs are not associated to any other breakpoints except their partner in the reciprocal pair, whereas breakpoints in non-reciprocal pairs can belong to two different pairs. Reciprocal pairs can result from simple inversions or translocations (see an example in Figure 7). On the other hand, non-reciprocal pairs can result from more complicated rearrangement events (like transpositions which involve three breakpoints) or from rearrangement series resulting from the re-use of a breakpoint by several rearrangements in the course of evolution (see an example in Figure 8).

We obtained 53 reciprocal breakpoint pairs on the human genome (containing 106 distinct breakpoints including 6 telomeres), and 276 non-reciprocal pairs containing 304 distinct breakpoints (including 37 telomeres).

### Assigning an evolutionary origin to breakpoints and breakpoint pairs

For each breakpoint region on the human genome, we tried to assign its evolutionary origin, that is to determine if the rearrangement occurred in the mouse or in the human lineage. To do so, we used the genome of the dog as an outgroup, and we identified human-dog and mouse-dog breakpoints using CASSIS.

If a human-mouse rearrangement is also observed between the human and dog genomes but not between the mouse and dog, then the most parsimonious scenario implies that the rearrangement took place in the human lineage. In the opposite case, the mouse lineage origin is the most parsimonious hypothesis.

More precisely, we call *Bh *the human-mouse breakpoint located on the human genome and *Bm*_1 _and *Bm*_2 _their corresponding breakpoints on the mouse genome. We assign a human lineage origin to *Bh *if i) it overlaps a human-dog breakpoint and ii) neither *Bm*_1 _nor *Bm*_2 _overlaps a mouse-dog breakpoint. On the contrary, we assign a mouse lineage origin to *Bh *if i) it does not overlap any human-dog breakpoint and ii)*Bm*_1 _and *Bm*_2 _overlap each a mouse-dog breakpoint. Otherwise, we assign no origin to the breakpoint.

We then assign an evolutionary origin to a breakpoint pair only if the two breakpoints of the pair have been assigned the same evolutionary origin. The criteria to assign an origin to a breakpoint pair are therefore quite strict, and they are more difficult to be met by non-reciprocal breakpoint pairs because of the re-use. In the end, we obtained 41 breakpoint pairs of human lineage origin (17 reciprocal and 24 non-reciprocal), 121 of mouse lineage origin (27 reciprocal and 94 non-reciprocal) and 167 of unknown origin (9 reciprocal and 158 non-reciprocal). The coordinates of breakpoint pairs on the human genome are provided in Additional File 2.

### Description of the HiC dataset

The data presented in [6] is available at the GEO database http://www.ncbi.nlm.nih.gov/geo/, accession number GSE18199. The raw data consist of millions of short sequence read pairs representing genomic loci that were in close contact in live human nuclei. The reads from each pair were independently mapped to the human genome using MAQ [6]. Each chromosome was divided in 1-Mb loci. The interaction between two loci is directly estimated by the number of read pairs where one read of the pair maps to one locus and the other read of the pair maps to the other locus. We restricted this analysis to the autosomal chromosomes and discarded loci covered by large assembly gaps (more than 50 percent of the sequence covered by N's). We obtained 2705 loci forming 3,659,865 locus pairs.

### Correcting for segmental duplications

**Figure 9:**
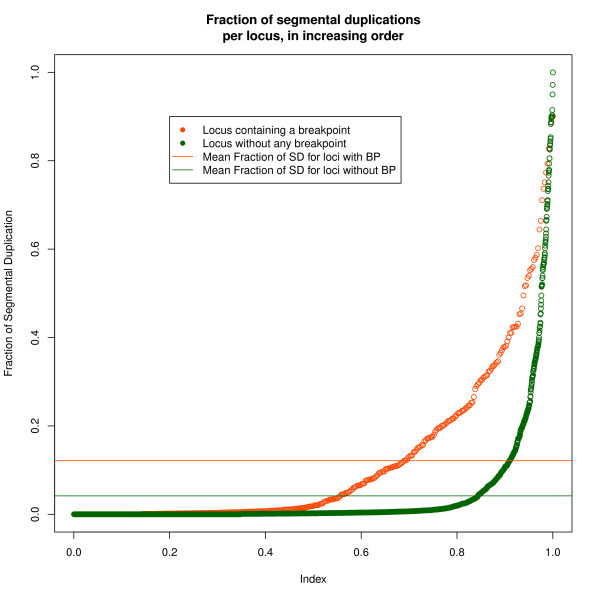
**Loci containing breakpoints are enriched in segmental duplications**. Loci containing breakpoints are enriched in segmental duplications.

Loci containing breakpoint regions are enriched in segmental duplications (see Figure 9). More than 40,000 segmental duplications are recorded in the human genome (UCSC human genome version 18), and each duplicated segment has a near identical DNA sequence. Mapping short reads of 35 bp to a unique location in the genome is made difficult by the presence of such repeated regions. When a read maps to several genomic locations, the software used to process the Hi-C reads randomly assigns the current read to one of the locations [9].

While this might be harmless when dealing with single reads in the context of genome (re-)sequencing, randomly choosing one of the copies of a segmental duplication induces a bias in the case of Hi-C paired-reads. Hi-C read pairs are ligation products from DNA fragments that were close to each other in the nucleus. Therefore, we expect that it will be more frequent for each read of a pair to map to genetically close regions, rather than to another segmental duplication copy in a remote location on the genome. In short, using MAQ to map short reads, the presence of segmental duplications within a locus could artefactually increase its number of long-distance Hi-C interactions due to a methodological problem during the mapping step.

Our proposed correction method relies on the assumption that the expected number of reads within a segmental duplication region is the same than the number of reads in the surrounding regions. The method can be described as follows:

• for each 1-Mb window, compute the fraction of the window occupied by sequences involved in segmental duplications (SDs)

• for each 1-Mb window pair (locus pair), compute the corrected number of reads for the locus pair (NRCSD) where , with

- NR: the number of pairwise reads from one locus to the other;

- SDR: the number of pairwise reads from one locus to the other, with at least one side of the read within a segmental duplication region;

- FSD1 and FSD2: the fraction of SDs in each of the two loci of the pair.

### Analysis of covariance

#### Genomic distance

In order to establish the link between spatial proximity, genomic distance and the presence of breakpoints, we considered the following models. Let *RC *be the frequency of interaction between loci, as estimated by the read count, corrected for repetitive sequences. Let *GD *be the genomic distance separating the locus pairs in Mb. Let *BP *be a boolean variable indicating whether the locus pair contains a breakpoint pair. For all models, the error is assumed to be normally distributed and the locus pairs are assumed to be independent:

• *M*_0 _: *log*(*RC_i_*) = *μ*_0 _+ *a*_0 _* *log*(*GD_i_*) + *e_i_*

• *M*_1 _: *log*(*RC_i_*) = *μ*_1 _+ *a*_1 _* *log*(*GD_i_*) + *b*_1 _* *BP_i _*+ *e_i_*

• *M*_2 _: *log*(*RC_i_*) = *μ*_2 _+ *a*_2 _* *log*(*GD_i_*) + *b*_2 _* *BP_i _*+ *c*_2 _* *log*(*GD_i_*) * *BP_i _*+ *e_i_*

We compared the models using Analysis of Covariance (ANCOVA) and showed that pairs of loci containing breakpoint pairs were spatially closer than pairs of loci not containing breakpoint pairs (*M*_1 _*vs*. *M*_0_, *p *= 4.74 * 10^-06^). We further found that, although this result is true for all distances, it is stronger for short distances (*M*_2 _*vs*. *M*_1_, *p *= 5.61 * 10^-13^).

The estimation of the parameters of the fitted models were the following:

5.808 <*μ*_0 _<*μ*_2 _<*μ*_1 _< 6.107, - 0.852 <*a*_1 _<*a*_2 _<*a*_0 _< - 0.776, *b*_1 _= 0.262, *b*_2 _= 0.728, *c*_2 _= -0.254.

#### Gene density and DNase sensitivity

Gene density in individual loci was computed as the sum of positions in the locus covered by genic parts over the length of the locus. Gene coordinates were taken from the "known genes" track of the UCSC genome browser [30] (on the hg18 human genome assembly). Gene density of a locus pair (*Gcov*) was measured as the product of the individual gene densities of each locus and was transformed in log scale to obtain a gaussian distribution (loci with gene density of 0 were discarded).

DNase sensitivity data were retrieved from the UCSC genome browser [31]. We selected the raw track of DNaseI produced by ENCODE in the same cell line as the Hi-C data (lymphoblastoid GM06990). This gives DNaseI cleavage densities along the chromosomes in sliding windows of 20 bp step. We then added these densities in each 1 Mb-locus. DNase sensitivity of a locus pair (*DNase*) was measured as the product of the individual DNase sensitivity of each locus and was transformed in log scale to obtain a gaussian distribution.

We then considered the following models:

• *M*_3 _: *log*(*RC_i_*) = *μ*_3 _+ *a*_3 _* *log*(*GD_i_*) + *b*_3 _* *log*(*Gcov_i_*) + *e_i_*

• *M*_4 _: *log*(*RC_i_*) = *μ*_4 _+ *a*_4 _* *log*(*GD_i_*) + *b*_4 _* *log*(*Gcov_i_*) + *c*_4 _* *BP_i _*+ *e_i_*

• *M*_5 _: *log*(*RC_i_*) = *μ*_4 _+ *a*_5 _* *log*(*GD_i_*) + *b*_5 _* *log*(*Gcov_i_*) + *c*_5 _* *BP_i _*+ *d*_5 _* *log*(*Gcov_i_*) * *BP_i _*+ *e_i_*

We compared the models using Analysis of Covariance (ANCOVA) and showed that pairs of loci containing breakpoint pairs are overall spatially closer than pairs of loci not containing breakpoint pairs (*M*_4 _*vs*. *M*_3_, *p *= 0.0234) and it is stronger in gene-rich regions (*M*_5 _*vs*. *M*_4_, *p *= 0.0298). The estimated parameters were the following: *μ*_3 _= 5.912, *μ*_4 _= *μ*_5 _= 5.911, *a*_3 _= *a*_4 _= *a*_5 _= -0.772, *b*_3 _= 0.0488, *b*_4 _= *b*_5 _= 0.0487, *c*_4 _= 0.135, *c*_5 _= 0.342, *d*_5 _= 0.129.

• *M*_6 _: *log*(*RC_i_*) = *μ*_6 _+ *a*_6 _* *log*(*GD_i_*) + *b*_6 _* *log*(*DNase_i_*) + *e_i_*

• *M*_7 _: *log*(*RC_i_*) = *μ*_7 _+ *a*_7 _* *log*(*GD_i_*) + *b*_7 _* *log*(*DNase_i_*) + *c*_7 _* *BP_i _*+ *e_i_*

• *M*_8 _: *log*(*RC_i_*) = *μ*_8 _+ *a*_8 _* *log*(*GD_i_*) + *b*_8 _* *log*(*DNase_i_*) + *c*_8 _* *BP_i _*+ *d*_8 _* *log*(*DNase_i_*) * *BP_i _*+ *e_i_*

We compared the models using Analysis of Covariance (ANCOVA) and showed that pairs of loci containing breakpoint pairs are spatially closer than pairs of loci not containing breakpoint pairs in loci of high DNaseI sensitivity (*M*_8 _*vs*. *M*_7_, *p *= 6.34 * 10^-11^). We notice that for loci with low DNAseI sensitivity, the effect is the opposite, resulting in a non detectable difference when we ignore the interaction between the breakpoint effect and DNAse sensitivity (*M*_7 _*vs*. *M*_6_, *p *= 0.608). The estimated parameters were the following: *μ*_7 _= 6.968, *μ*_8 _= 6.965, *a*_7 _= *a*_8 _= -0.778, *b*_7 _= *b*_8 _= 0.189, *c*_7 _= 0.030, *c*_8 _= 1.68, *d*_8 _= 0.308. Finally, we also verified that the interaction between genomic distance and gene density or between genomic distance and DNAse sensitivity were not confounding factors for the breakpoint effect. We outline that our purpose in this work is not to find a full model that best describes the data, but simply to test if there is a difference between pairs of loci containing or not containing breakpoint pairs. The other factors are seen as possible confounding factors which we need to control for.

### Simulations

In all the tests we previously performed, we worked under the assumption that the variables under consideration are independent, normally distributed, and that the distributions to be compared have an equal variance. Even though we checked that these working hypotheses were reasonable, these assumptions are never strictly met. In particular, the tail of the distribution deviates from a Gaussian distribution. We therefore also tested our hypotheses using simulations, which do not require any of the hypotheses mentioned above.

The simulations compare the mean number of reads for locus pairs containing a breakpoint pair to the mean number of reads of random sets of locus pairs not containing a breakpoint pair. To take into account the known biases, the locus pairs were partitioned into classes of genomic distance, gene density and DNase sensitivity. In each simulation, the number of locus pairs containing a breakpoint pair is recorded for each category. The same number of locus pairs is then randomly picked within the set of locus pairs not containing a breakpoint pair and fitting within the category. The mean number of reads for the picked pairs is then recorded. For each simulation, this process is repeated 500 times.

We created 9 classes of distance of the same length in the logarithmic scale plus one additional class for the inter-chromosomal pairs. The gene density of a locus pair was measured by the product of the gene density of each locus. Consequently, 4 classes were defined with the following thresholds (0, 0.1, 0.25, 1), representing pairs without any genes, pairs with a low density, medium density and high density of genes. We set two levels of DNase sensitivity: rich and poor. Consequently, we defined three classes of DNase sensitivity locus pairs: rich-rich, rich-poor and poor-poor.

We first only used the distance classes, then used both distance and gene density (a total of 40 classes), and then both distance and DNase sensitivity (30 classes).

We further checked that the results hold whether we use all locus pairs or only the intra-chromosomal pairs. However, inter-chromosomal pairs containing breakpoints are not significantly more often found in contact than those without breakpoints.

## Authors' contributions

AV collected the data from [6] while CL prepared the data on breakpoint rearrangements using Cassis [5,7]. AV, CL and VL conducted the computational analyses described in the paper. All authors contributed equally to conceiving the experiments and writing the manuscript. All authors read and approved the final manuscript.

## Supplementary Material

Additional file 1 **Supplementary Figures**. This file contains the supplementary figures mentioned in the text. Click here for file

Additional file 2 **Breakpoint pair coordinates**. Plain text file containing all breakpoint pair coordinates on the human genome. Coordinates correspond to human assembly of November 2005 (NCBI36 or hg18). Additional columns indicate their evolutionary origin (human, mouse or unknown), their type (R for reciprocal and NR for non-reciprocal) and if the pair was used in the present analysis (yes or no). Click here for file
