# Supplementary material for: Close 3D proximity of evolutionary breakpoints argues for the notion of spatial synteny
Source: BMC Genomics. 2011 Jun 10;12:303. doi: 10.1186/1471-2164-12-303 (PMC3132170; doi:10.1186/1471-2164-12-303)
Supplement: Additional file 1 — Supplementary Figures. This file contains the supplementary figures mentioned in the text. [file 1471-2164-12-303-S1.PDF]

Additional File 1 for

Close 3D proximity of evolutionary breakpoints argues for the notion of spatial synteny

Amélie S Véron[*1,2,3,4], Claire Lemaitre[*5,6], Christian Gautier[1,2,3], Vincent Lacroix[*1,2,3] and Marie-France Sagot[*1,2,3]

[1] *Université de Lyon, F-69000 Lyon, France*
[2] *Laboratoire Biométrie et Biologie Evolutive, CNRS,Université Lyon 1, F-69100 Villeurbanne, France*
[3] *Equipe BAMBOO, INRIA Grenoble Rhône-Alpes, 655 avenue de l'Europe,F-38330 Montbonnot Saint-Martin, France*
[4] *INSERM U1052, Cancerology Research Center of Lyon, Centre Léon Bérard, Lyon, France*
[5] *Université de Bordeaux, Centre de Bioinformatique et Génomique Fonctionnelle Bordeaux, F-33000 Bordeaux, France*
[6] *Equipe SYMBIOSE, INRIA Rennes Bretagne Atlantique, Campus de Beaulieu, F-35042 Rennes, France*
[*] *Corresponding authors : AV: amelie.veron@gmail.com, CL: claire.lemaitre@inria.fr, VL: Vincent.Lacroix@univ-lyon1.fr, MFS: Marie-France.Sagot@inria.fr*

Figure S1 - Overview of the number of reads per locus pair at a given distance.

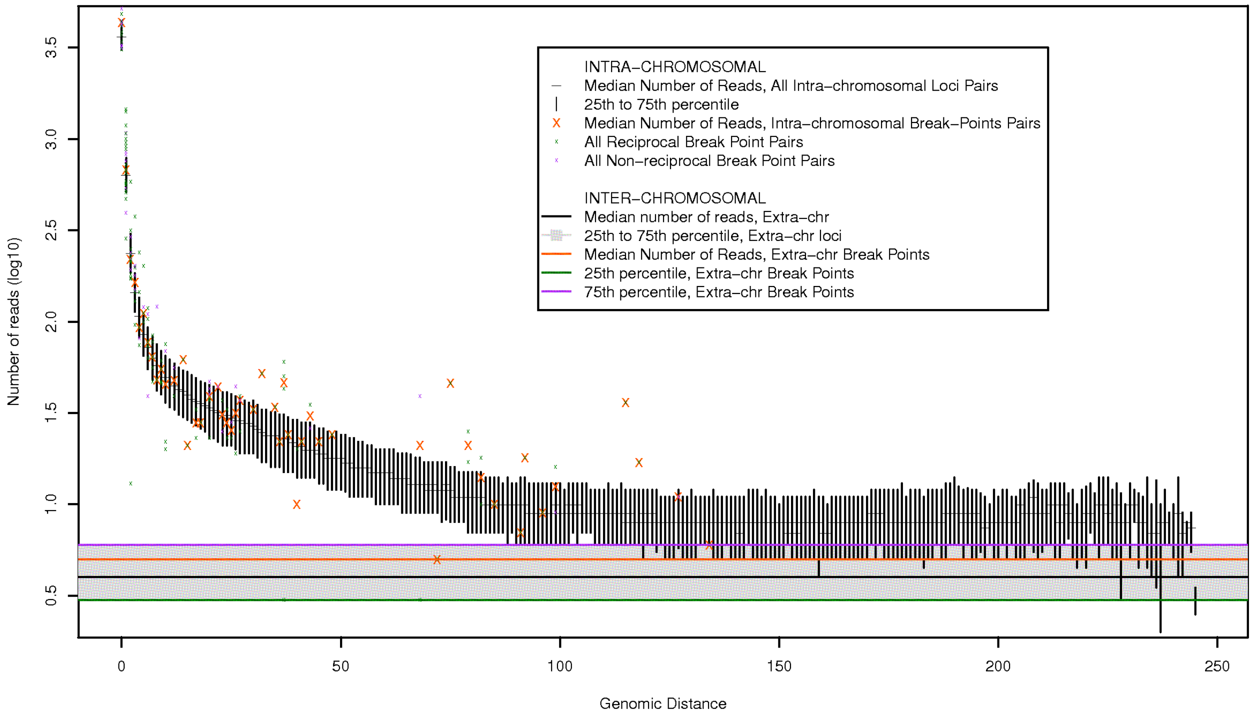

Figure S1: Overview of the number of reads per locus pair at a given distance. The median, 25th and 75th percentile are shown for all loci. For the breakpoint loci, only the me dian is shown (there is often only one data point per distance). Horizontal lines display the values for inter-chromosomal data. From this figure, it is clear that the inter- chromosomal contacts are much less frequent than even long-range intra-chromosomal contacts.

Figure S2 - Read count for locus pairs less than 1 Mb apart

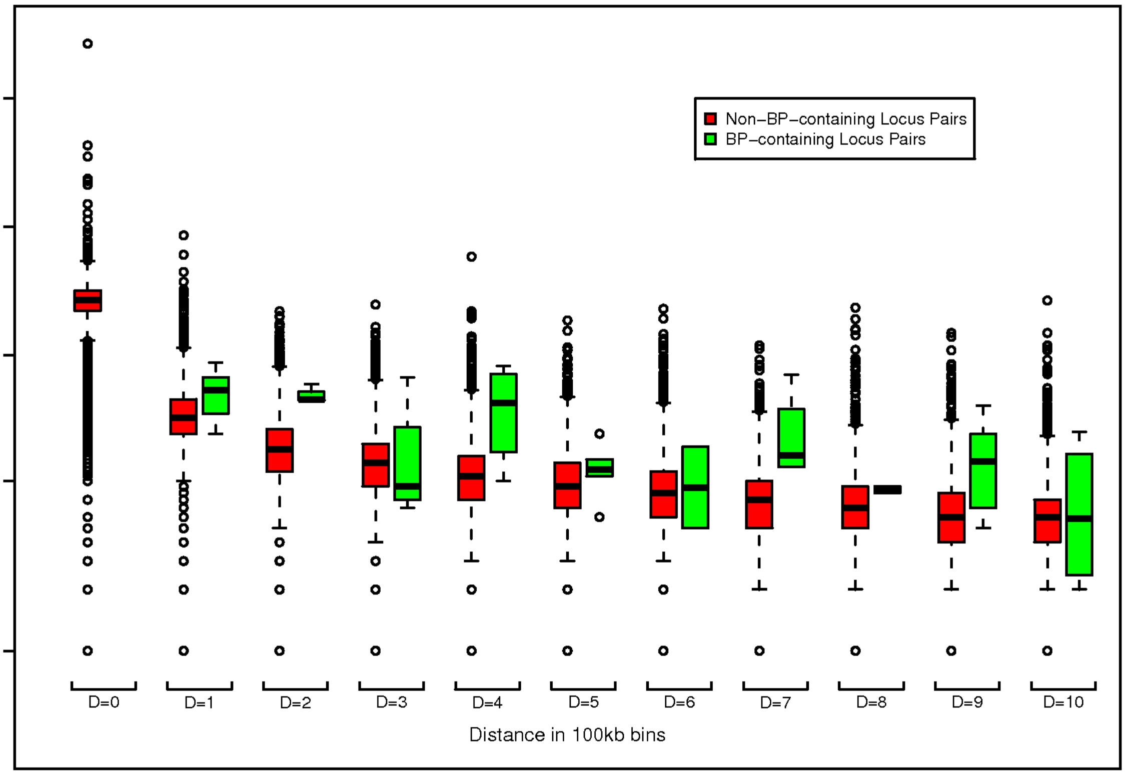

Figure S2: Read count for locus pairs less than 1 Mb apart (loci bins of 100kb).

Figure S3 - Read count for locus pairs containing a breakpoint pair or not in several classes of gene density

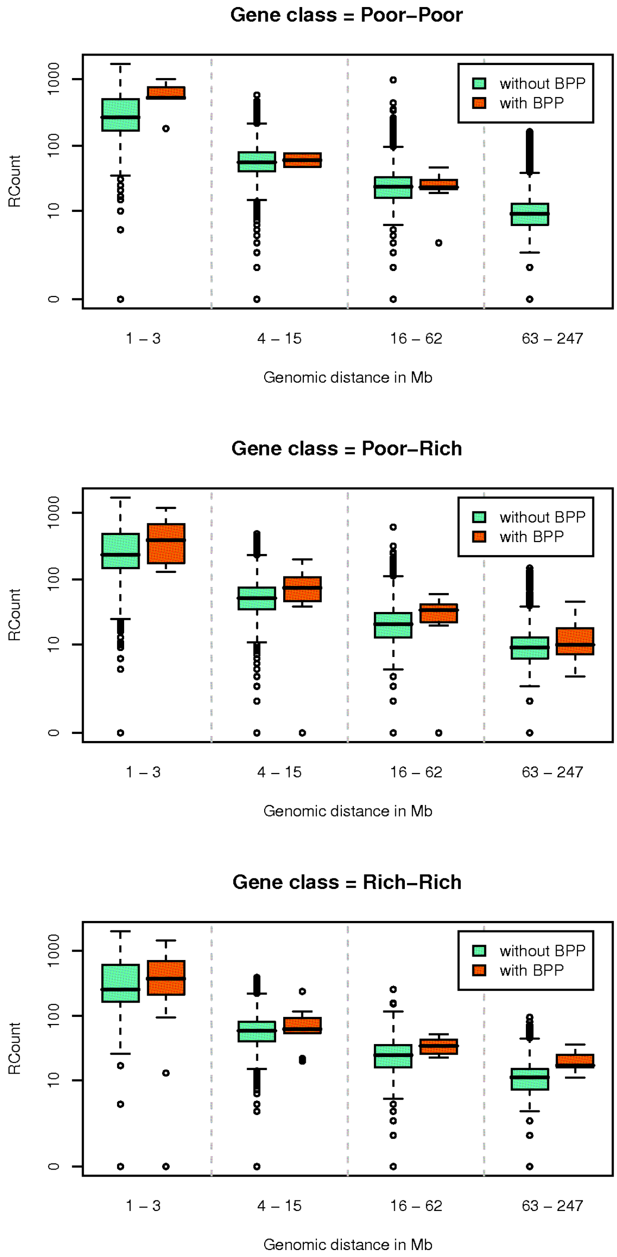

Figure S3: Each figure represents the frequency of interaction (read counts) of locus pairs containing or not breakpoint pairs in several classes of genomic distances, in a given class of gene density. Loci were classified in two classes of equal size depending on their genic coverage : "poor" if their genic coverage is less than 45 %, "rich" otherwise. Locus pairs were then classified in three classes as follows : "poor-poor" (resp. "rich-rich") if both loci belongs to the "poor" (resp. "rich") class, "poor-rich" if the two loci belongs to different gene density classes. The read count is corrected for the presence of segmental duplications.

Figure S4 - Read count for locus pairs containing a breakpoint pair or not in several classes of DNaseI sensitivity

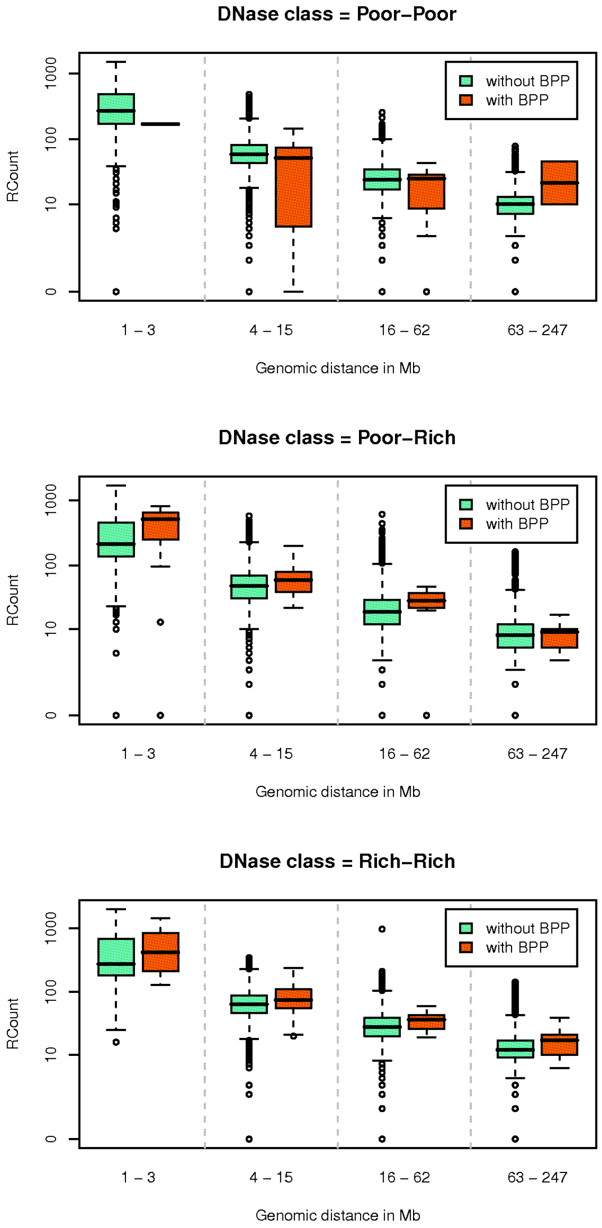

Figure S4: Each figure represents the frequency of interaction (read counts) of locus pairs containing or not breakpoint pairs in several classes of genomic distances, in a given class of DNase sensitivity. Loci were classified in two classes of equal size depending on their DNase sensitivity : "poor" for the less sensitive (sum of intensities below 48000), "rich" otherwise. Locus pairs were then classified in three classes as follows : "poor-poor" (resp. "rich-rich") if both loci belongs to the "poor" (resp. "rich") class, "poor-rich" if the two loci belongs to different DNase sensitivity classes. The read count is corrected for the presence of segmental duplications.
